# Gene expression profiles underlying aggressive behavior in the prefrontal cortex of cattle

**DOI:** 10.1186/s12864-021-07505-5

**Published:** 2021-04-07

**Authors:** Paulina G. Eusebi, Natalia Sevane, Thomas O’Rourke, Manuel Pizarro, Cedric Boeckx, Susana Dunner

**Affiliations:** 1 Universidad Complutense de Madrid, Avenida Puerta de Hierro, s/n, 28040 Madrid, Spain; 2 Universitat de Barcelona, Gran Vía de les Corts Catalanes 585, 08007 Barcelona, Spain; 3 UBICS, Carrer Martí Franqués 1, 08028 Barcelona, Spain; 4 ICREA, Passeig Lluís Companys 23, 08010 Barcelona, Spain

## Abstract

**Background:**

Aggressive behavior is an ancient and conserved trait, habitual for most animals in order to eat, protect themselves, compete for mating and defend their territories. Genetic factors have been shown to play an important role in the development of aggression both in animals and humans, displaying moderate to high heritability estimates. Although such types of behaviors have been studied in different animal models, the molecular architecture of aggressiveness remains poorly understood. This study compared gene expression profiles of 16 prefrontal cortex (PFC) samples from aggressive and non-aggressive cattle breeds: Lidia, selected for agonistic responses, and Wagyu, selected for tameness.

**Results:**

A total of 918 up-regulated and 278 down-regulated differentially expressed genes (DEG) were identified, representing above-chance overlap with genes previously identified in studies of aggression across species, as well as those implicated in recent human evolution. The functional interpretation of the up-regulated genes in the aggressive cohort revealed enrichment of pathways such as Alzheimer disease-presenilin, integrins and the ERK/MAPK signaling cascade, all implicated in the development of abnormal aggressive behaviors and neurophysiological disorders. Moreover, gonadotropins, are up-regulated as natural mechanisms enhancing aggression. Concomitantly, heterotrimeric G-protein pathways, associated with low reactivity mental states, and the *GAD2* gene, a repressor of agonistic reactions associated with PFC activity, are down-regulated, promoting the development of the aggressive responses selected for in Lidia cattle. We also identified six upstream regulators, whose functional activity fits with the etiology of abnormal behavioral responses associated with aggression.

**Conclusions:**

These transcriptional correlates of aggression, resulting, at least in part, from controlled artificial selection, can provide valuable insights into the complex architecture that underlies naturally developed agonistic behaviors.

This analysis constitutes a first important step towards the identification of the genes and metabolic pathways that promote aggression in cattle and, providing a novel model species to disentangle the mechanisms underlying variability in aggressive behavior.

**Supplementary Information:**

The online version contains supplementary material available at 10.1186/s12864-021-07505-5.

## Background

Aggression, an evolutionary well-conserved trait, is part of the behavioral repertoire across species, as most animals need this skill in order to eat, protect themselves and their families against predators, compete for mates, and acquire resources and territory [1]. In contrast, scientific interest in human aggression is often centered on abnormal manifestations of the behavior, including violence associated with dementias or neuropsychiatric disorders, such as manic depression, bipolar disorder, schizophrenia, as well as conduct and antisocial personality disorders [2, 3]. Research has shown that the expression of aggressive behavior depends on the interaction between environmental and genetic factors, with a genetic additive component ranging around 50% in humans [4].

A large number of preclinical studies using different animal species as models has been encouraged on the reasoning that molecular correlates of animal aggressive behaviors resemble varying biological mechanisms in human pathological aggression [5]. Several attempts have been made to mold abnormal forms of aggressiveness, mainly using murine models, and to a lesser extent dogs and semi-domesticated species such as the silver fox, in order to display a contrast between docile or tame behaviors and escalated levels of aggressiveness [6]. However, relating these mechanisms to the human condition is not simple, given the polygenic basis and diverse instantiations of aggressive behaviors. In animals, aggressive responses consist of a combination of fight, chase, bite and ram, whereas aggression in humans involves both verbal and physical forms. Despite this, the identification of similar components of aggression across species can help to better understand its etiology and to further improve its diagnosis, prognosis and intervention strategies, which currently lack in effectiveness [7].

Domesticated species offer particularly interesting models for research into human aggression. Over recent years, genomic, transcriptomic, behavioral, and archaeological evidence has begun to accumulate, indicating that anatomically modern humans and domesticated species have followed convergent evolutionary processes compared to their respective archaic and wild counterparts [8–10]. Our species exhibits craniofacial alterations reminiscent of those typical in the “domestication syndrome”, including reduced tooth size, contraction of the skull, and flattening of the face (comparable to the shortened muzzles of domesticates) [11]. The Russian farm-fox experiment has shown that such broad phenotypical changes can emerge from selection for reduced reactive aggression towards humans, a trait ubiquitous across domesticated species [12]. In conjunction with findings that our species has markedly reduced intraspecific reactive aggression when compared to extant primates, this has helped to spur research into the hypothesis that, relative to archaic hominins, modern humans have undergone positive selection for a reduction in reactive aggression towards each other [13].

Similarly to farm foxes selected for aggressive behaviors, a reduction in reactive aggression is exceptionally absent in the case of the Lidia breed of cattle. Lidia bovines belong to a primitive population, selected for centuries to develop agonistic-aggressive responses by means of a series of traits that are registered by breeders on a categorical scale, which classifies aggression and fighting capacity, reporting moderate to high heritability estimates for the Lidia (0.20–0.36) [14, 15]. Thus, within the bovine species, Lidia cattle may constitute a useful tool for studying the genomic makeup of aggressive behavior. The utility of cattle as a model for human aggression is further underscored by exploratory findings that selective sweeps implicated in cattle domestication have above-chance intersection with those identified in modern humans relative to archaics [10]. A recent study has identified significant divergence in genomic regions containing genes associated with aggressive behavior in the Lidia breed [16]. This includes a polymorphism in the promoter of the monoamine oxidase A (*MAOA*) gene, an important locus widely associated with pathological forms of aggression which, in humans, manifests in a broad spectrum of psychiatric conditions, such as manic and bipolar disorders and schizophrenia, among others [17, 18]. Similarly, the kainite glutamate receptor *GRIK3* is associated with heightened aggression in Lidia cattle. This gene has been targeted in modern human evolution and in multiple domestication events, including in dogs, sheep, yaks, and across multiple cattle breeds [16, 19, 20]. However, no studies on gene expression differences for behavioral features have been conducted so far in cattle.

Gene expression in the prefrontal cortex (PFC) has been shown to play a crucial role in the regulation of aggressive behavior [21, 22]. The PFC role in aggression has been studied in different species, e.g. PFC lesions result in impulsive and antisocial behaviors in humans [23] and offensive aggression in rodents [17]. Moreover, a catalogue of gene-specific sequence variants was detected as differentially expressed between a strain of silver fox selected for aggressive behaviors when compared to its tame counterpart [24]. Similar results are reported in RNA-seq profiles of different dog breeds [25].

The goal of our study is to uncover genes that are differentially expressed in the PFC of aggressive and non-aggressive bovines using as models the Lidia and the Wagyu breeds as aggressive and non-aggressive cohorts respectively. The two breeds differ significantly in their agonistic responses, the Lidia being known as one of the most aggressive bovine breeds, whereas Wagyu bovines are docile animals, selected and bred by farmers with the aim of easing their handling [26]. These divergent phenotypes, in conjunction with the potential relevance of domestication events to recent human evolution, make our populations of study as suitable for research into the biological underpinnings of aggressive behavior in animals, as well as abnormal aggression in humans.

## Methods

This study did not involve purposeful killing of animals, thus, no special permits were required to conduct the research. Samples were collected from bovines after slaughter following standard procedures approved by the Spanish legislation applied to abattoirs [27]. No ethical approval was deemed necessary.

### Animals, sample retrieval and tissue processing

Post mortem PFC tissue samples were collected (in May 2019) from 16 non-castrated male bovines aged 3 to 4 years, 8 belonging to the Lidia breed, considered aggressive (*n* = 8), and 8 belonging to the Wagyu breed, considered tamed (n = 8). Animals from the aggressive Lidia group belong to two batches: one from “La quinta” farm (*N* = 4, coordinates: 37°44′39″N 5°17′32″O) and the other from “Montealto” farm (N = 4, coordinates: 40°49′35″N 3°38′30″W) (Supplementary Table 1), both affiliated to the Lidia Breeders Association (UCTL, https://torosbravos.es/), whose genealogical and behavioral data have been previously studied and recorded [15]. From the docile cohort, the batch of 8 Wagyu bulls belong to the farm “Nuestro Buey” (https://www.fincasantarosalia.com/, coordinates: 42°16′24″N 4°09′23″W) and were raised exclusively for meat production purposes.

The study is designed on the basis of the differences in aggressiveness reached through intensive human selection over the last centuries; whereas the Lidia breed has been selected exclusively for aggressive behavior related traits, the tamed Wagyu breed has been selected for meat quality traits and docile behaviors in order to facilitate handling [26]. Among the wide variety of docile cattle breeds, we opted for the Wagyu breed due to its age at slaughter, higher than 36 months, like that of Lidia breed bulls [28].

All of the selected Lidia individuals belonged to an “elite” group of aggressive bulls, selected by their breeders according to the standardized traits of aggressiveness, ferocity, face hiding and nobility on a categorical scale from 1 to 10 for each trait [14, 28]. The genealogical and behavioral scores of these traits have been recorded between 1984 and 2010 and analyzed by Menéndez-Buxadera et al. [15], using multi-trait reaction norm models, which revealed heritability values ranging between 0.230 and 0.308, with aggressiveness attaining the highest heritability score.

Non-related Lidia individuals were raised under an extensive farming system, pasture fed until 6–8 months prior to their sacrifice. At this stage bulls were separated into wide-fenced enclosures and fed with a fattening supplementary diet of ad-libitum high energy and highly digestible concentrates [29]. The Wagyu cattle handling practices are to raise animals freely grazing within the farm’s pastures at a young age to produce quality meat that satisfies consumer preferences and reduce production costs. From 11 months until their sacrifice, the animals are fed with a high-concentrate diet (ad-libitum) to induce higher intramuscular fat [30].

Prior to the “corrida” event, the Lidia bovines were incited to develop agonistic-aggressive behaviors, with their performance measured, based on the four traits defined above. The eight individuals displayed similar scores (Supplementary Table 2). The Wagyu bovines were handled in the same batch as they were reared in and were transported together to the slaughterhouse, which entails inherent stress to them; as expected from their natural docile behavior, no agonistic encounters were registered among them nor against the personnel at the slaughterhouse.

PFC samples from the Lidia and Wagyu bulls were taken at the Plaza de Toros and slaughterhouse cutting rooms, respectively. To retrieve the PFC samples, the same method was used in all cases: skulls were cut in a transverse plane into dorsal and ventral halves to expose the brains. Samples from the right half of the dorsal brain of each bull were used for the transcriptomic study (Figure S1) harvesting PFC tissue samples (0.2 -0.3 gr) from both cohorts less than 1 h *post-mortem*. Sampling was performed with unaided eye by the same person and by using a set of sterilized and autoclaved scalpels and tissue scissors. The collection of samples was recorded using photographs and anatomical location of the sequenced brain regions is presented in Figure S1. Samples were immediately immersed in RNA-later™ (Thermo Fisher Scientific, Madrid, Spain), followed by 24-h storage at 5 °C and long-term conservation at − 80 °C.

### RNA extraction, sequencing and bioinformatics analyses

Total RNA was extracted from postmortem PFC tissue using the RNeasy Lipid Tissue Mini Kit (QIAGEN, Spain) according to the manufacturer’s instructions. Tissuelyser (QIAGEN, Spain) was used to homogenize samples. RNA quantification and purity were assessed with a Nanodrop ND-1000 spectophotometer (Thermo Fisher Scientific, Madrid, Spain) and RNA integrity number (RIN) was determined using the Bioanalyzer-2100 equipment (Agilent Technologies, Santa Clara, CA, USA). To guarantee its preservation, RNA samples were treated with RNAstable (Sigma-Aldrich, Madrid, Spain), and shipped at ambient temperature to the sequencing laboratory (DNA-link Inc. Seoul, Korea) to perform high throughput sequencing using a Novaseq 6000 sequencer (Illumina, San Diego, CA, USA). For quality check, the OD 260/280 ratio was determined to be between 1.87 and 2.0. Library preparation for Illumina sequencing was done using the Illumina Truseq Stranded mRNA Preparation kit (Illumina, San Diego, CA, USA). Sequencing was performed in 100 base paired-end mode, followed by automatic quality filtering following Illumina specifications. All these processes were performed according to the manufacturer’s instructions. Individual reads were de-multiplexed using the CASAVA pipeline (Illumina v1.8.2), obtaining the FASTQ files used for downstream bioinformatics analysis.

Read quality of the sixteen RNA-seq datasets was checked and trimmed using PRINSEQ v. 0.20.4 [31]. Trimmed reads were then mapped to the bovine reference genome (*Bos taurus* ARS.UCD 1.2) with STAR v.2.7.3a [32], using default parameters for pair-end reads and including the Ensembl *Bos taurus* ARS-UCD 1.2 reference annotation. The SAM files generated by STAR, which contains the count of reads per base aligned to each location across the length of the genome, were converted into a binary alignment/map (BAM) format and sorted using SAMTools v.0.1.18 [33]. The aligned RNA-seq reads were assembled into transcripts and their abundance in fragments per kilobase of exon per million fragments mapped (FKPM) was determined with Cufflinks v.2.2.1 [34, 35]. The assembled transcripts of all samples were merged using the Cufflinks tool “Cuffmerge”. Analysis of differential gene expression across aggressive and non-aggressive groups was performed using Cuffdiff, included also in the Cufflinks package. A Benjamini-Hochberg False Discovery Rate (FDR), which defines the significance of the Cuffdiff output, was set as threshold for statistically significant values of the Differentially Expressed Genes (DEG). The R software application CummeRbund v.2.28.0 [36] was used to visualize the results of the RNA-seq analysis.

### Cross-species comparative analysis (CSCA)

Because no other differential expression analysis using cattle as an animal model for aggressive behaviors has been conducted before, we performed a comparison between our DEG and a cross-species compendium of genes associated with aggressiveness previously identified in different studies in humans, rodents, foxes, dogs and cattle, as proposed by Zhang-James et al. [37]. The gene-set compendium is a list based on four main categories of genetic evidence: i) two sets of genes identified in different genome-wide association studies (GWAS) in humans, one for adults and the other for children [38]; ii) one set of genes showing selection signatures in Lidia cattle [16, 18]; iii) four sets of genes differentially expressed in rodents [39, 40] and one in silver foxes [24, 41]; and iv) three sets of genes with causal evidence from the Online Mendelian Inheritance in Man (OMIM) database, a knockout (KO) mice report and causal evidence in dogs retrieved from the Online Mendelian Inheritance in Animals (OMIA) database [25, 36].

To homogenize the compendium gene-list with our DEG, gene official names from cattle were converted to their human orthologues using biomaRt [41]. In order to establish a ranking according to the total occurrence of each gene in the different sets, we assigned a weight (weighted ranking, WR) to each of our DEG in common with the compendium gene list, applying the same conditions proposed by Zhang-James et al. [36].

For statistical analysis of the intersection between our DEG and genes identified in different studies of aggression, we cross-referenced each gene list using Panther v.12.0 (www.pantherdb.org), NCBI HomoloGene,(www.ncbi.nlm.nih.gov/homologene) and Ensembl orthologue databases with the *Bos taurus* ARS-UCD 1.2 and Human reference (GRCh38.p13) genomes. If no human–bovine one-to-one orthologues were found in any database, we removed the relevant genes for statistical analysis. The compendium gene-list can be found in Supplementary Table 3.

To evaluate the possibility that Lidia divergence from the domesticated transcriptional profile of the Wagyu follows a similar pattern to divergence between archaic and modern humans, we compared the intersection of Lidia DEGs with genes containing disproportionate rates of high-frequency mutations in archaic compared modern humans and vice-versa. These included comparisons with genes harboring excess mutations, excess missense mutations, and excess mutations in regulatory regions. We also compared the Lidia DEGs with genes targeted by selective sweeps in modern human and domesticate evolution. These distinct gene lists (thirteen in total) are compiled by Zanella et al. 2019 [42] (Supplementary Table 4).

### Gene ontology and KEGG pathway enrichment analyses

To examine the relationships between differences in PFC gene expression among groups and their biological functions, the Log_2_ Signal Fold Change (FC) score was used to partition the DEG into up-regulated and down-regulated groups. The Panther database v.12.0 was then used to determine processes and pathways of major biological significance through the Over Representation test based on the Gene Ontology (GO) annotation function. Panther applies different algorithms using the uploaded reference lists as seeds and known interactions from the database as edges to generate content specific pathways. We used Fisher’s exact test for annotation and the FDR for multiple testing corrections, both for the up and down regulated DEG with *P*-values ≤0.05, to infer their pathway enrichment scores.

### Biological role of the genes in common with the CSCA: interactions and upstream regulators

The Ingenuity Pathway Analysis (IPA) (QIAGEN, www.qiagen.com/ingenuity) software was used to identify GOs, pathways and regulatory networks to which our DEG in common with the compendium gene-list belong, as well as these genes’ upstream regulators; a threshold of WR values greater than or equal to 1 was set for the DEG in common with the CSCA in order to restrict the analysis to the most significant genes within the compendium gene-set. IPA transforms a set of genes into a number of relevant networks based on comprehensive records maintained in the Ingenuity Pathways Knowledge Base. The networks are presented as graphics depicting the biological relationships between genes and gene products. The analysis of upstream regulators considers all possible transcription factors, as well as their predicted effects on gene expression contained in the Base repository. Therefore, IPA enables analysis of whether the patterns of expression observed in the DEG can be explained by the activation or inhibition of any of these regulators through an estimation of a z-score, a statistical measure of the match between the expected directional relationship between the regulator and its targets, based on observed gene expression [43].

## Results

### Sequencing and read assembly

**Fig. 1:**
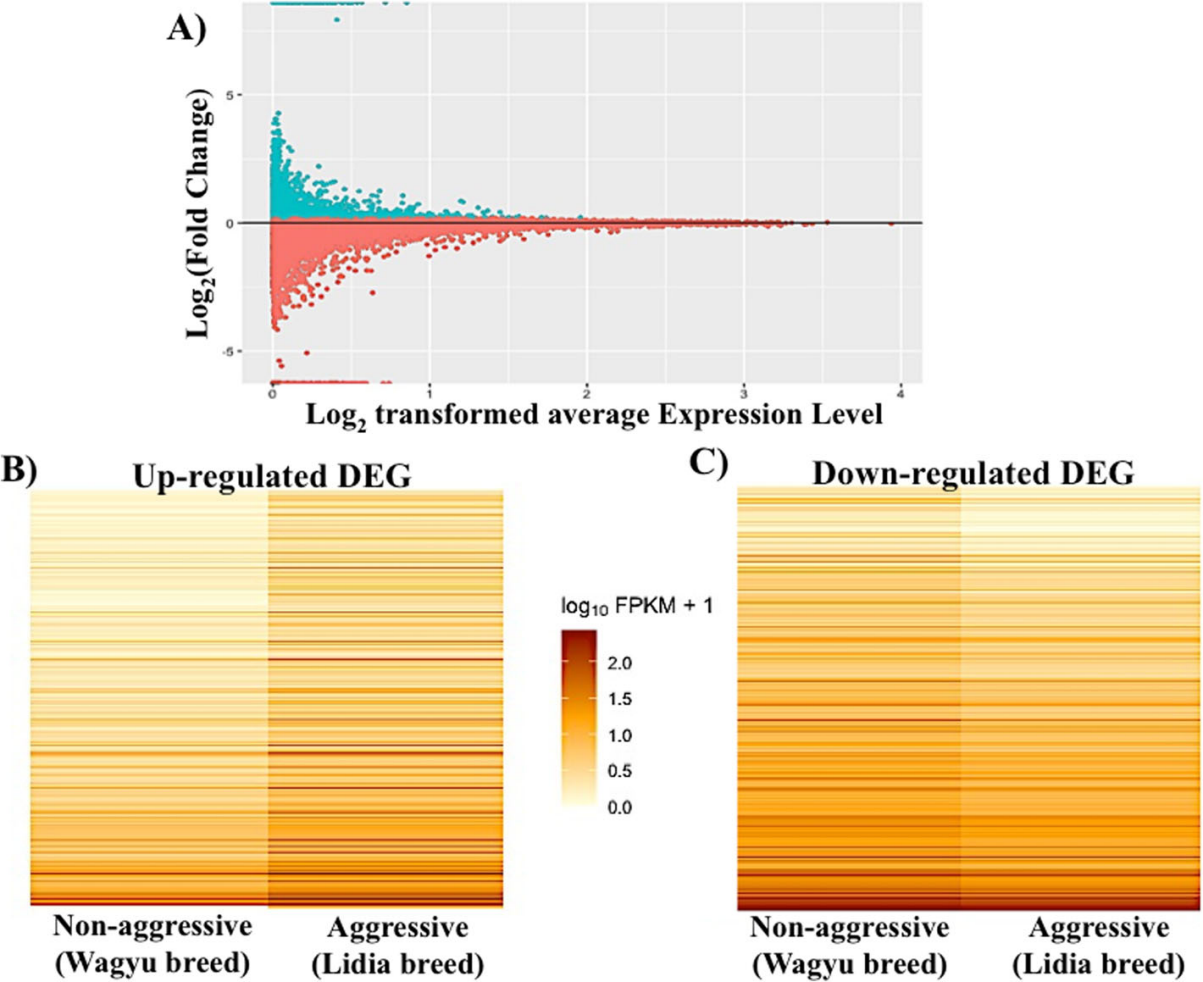
**a** MA-plot showing the distribution of differentially expressed genes (DEG). The Y-axis shows the log_2_ (Fold Change) of expression between aggressive and non-aggressive groups, and the X-axis corresponds to the log_2_ transformed average expression level for each gene across samples. Log_2_FC ≥ 0.1 and Log_2_FC ≤ 0.1 genes are represented by green and red dots, respectively. **b** Heatmap of up-regulated DEG in the aggressive group. **c** Heatmap of down-regulated DEG in the aggressive group

The RNA-sequencing of the sixteen PFC samples generated an average of 78.3 million paired-end reads per sample. The mean proportion of mapped reads with the STAR software was 91.8%, similar among different samples (from 88.07 to 94.91%) (Supplementary Table 1). The mapped reads were processed with Cufflinks toolkits for differential expression analysis, revealing a total of 16,384 DEG between the aggressive and non-aggressive groups; of these genes, 1196 were statistically significant, producing 10,640 isoforms (8.86 transcripts per gene) (Table 1, Fig. 1a). Gene expression differences of the up-regulated DEG (log_2_FC ≥ 0.1) were greater in number, involving 918 genes, than those down-regulated; 278 DEG (log_2_FC ≤ 0.1) (Fig. 1b and c). For the complete list of up and down-regulated DEG see Supplementary Table 5.

**Table 1:** Summary statistics of differentially expressed features

Classification	Transcripts
Significant DEG	1196
Up-regulated DEG	918
Down-regulated DEG	278
Differentially expressed isoforms	10,640

### Genes in common with the cross-species comparative analysis (CSCA)

The up and down-regulated DEG ≥1 WR values were compared with the compendium gene-list associated with aggressive behavior (Supplementary Table 3). This comparison yielded 50 genes, 24 up and 26 down-regulated in the aggressive group of Lidia individuals (Table 2).

**Table 2:** Up and down regulated DEG in common with the cross-species comparative analysis (CSCA)

**UP-REGULATED**		
**Gene symbol**	**Gene name**	**Weighted Ranking (WR)**
*SCD*	Stearoyl-CoA Desaturase	2.5
*LAMA2*	ADAM metallopeptidase with thrombospondin type 1 motif 1	2
*DRD2*	Dopamine receptor 2	1.5
*DUSP1*	Dual specificity phosphatase 1	1.5
*ANTRXR2*	ANTXR Cell Adhesion Molecule 2	1
*FGFR1*	Fibroblast growth factor receptor 1	1
*PDLIM4*	PDZ and LIM domain 4	1
*PNRC2*	Proline rich nuclear receptor co-activator 2	1
*SCARA5*	Scavenger Receptor Class A member 5	1
*RAB3IL1*	RAB3A Interacting Protein Like 1	1
*H3F3A*	H3.3 Histone A	1
*PAMR1*	Peptidase domain containing associated with muscle regeneration 1	1
*ADAMTS1*	ADAM metallopeptidase with thrombospondin type 1 motif 1	1
*ZAP70*	Zeta Chain of T Cell Receptor Associates Protein Kinase 70	1
*COL13A1*	Collagen type XIII alpha chain 1	1
*DACT2*	Dishevelled binding antagonist of beta catetin 2	1
*EPHX1*	Epoxide hydrolase 1	1
*EVC2*	EvC cillary complex subunit 2	1
*EYA2*	EYA Transcriptional co-activator and phosphatase 2	1
*ZNF786*	Zinc Finger protein 786	1
*RARRES1*	Retinoic Acid Receptor Responder 1	1
*SOX17*	SRY-Box Transcription Factor 17	1
*TOX*	Thymocyte Selection Associated High Mobility Group Box	1
*IGF2*	Insulin like growth factor 2	1

**DOWN-REGULATED**		
**Gene symbol**	**Gene name**	**Weighted Ranking (WR)**
*GAD2*	Glutamate decarboxylase 2	2
*DNAJB5*	Dnaj Heat shock protein family (Hsp40) member B5	2
*PNOC*	Propionociceptin	2
*RIMBP2*	RIMS Binding Protein 2	2
*ADCYAP1*	Adelynate cyclase activating polypeptide 1	1.5
*BDNF*	Brain derived neurotropic factor	1.5
*BCL2L1*	BCL2 Like 1	1.5
*CNR1*	Cannabioid Receptor 1	1.5
*CRHBP*	Corticotropin releasing hormone binding protein	1.5
*DGKG*	Diacylglycerol Kinase Gamma	1.5
*EGR3*	Early growth response 3	1.5
*HOMER1*	Homer scaffold protein 1	1.5
*PAK3*	P21 (RAC1) activated kinase 3	1.5
*CBLN1*	Cerebelin 1 Precursor	1.5
*SLC24A2*	Solute carrier family 24 member 2	1
*VGF*	VGF nerve growth factor inducible	1
*CDH13*	Cadherin 13	1
*PDE4D*	Phosphodiesterase 4D	1
*ARHGEF3*	Rho Guanine Nucleotide Exchange Factor 3	1
*COL4A1*	Collagen Type IV Alhpa I Chain	1
*FSTL5*	Follistatin Like 5	1
*JAZF1*	JAZF Zinc Finger 1	1
*ISM1*	Isthmin 1	1
*ZBTB16*	Zinc FB Finger and BTB Domain Containing 16	1
*ARHGAP25*	Rho GTPase Activating Protein 25	1
*SEL1L3*	SEL1L Family Member 3	1

### Functional annotation and biological pathway analysis

A GO analysis of the pathways and biological processes identified in the dataset lists containing significant up and down-regulated transcripts was carried out. Among the 918 up-regulated DEGs in aggressive Lidia samples, Panther Over Representation test included 851 uniquely mapped IDs, displaying significant association with 881 GO biological processes (FDR ≤ 0.05), most of them related to heart morphogenesis and heart development, cellular adhesion, migration and differentiation, skeletal and smooth muscle development, central nervous system (CNS) development and function, and immune response (Supplementary Table 5). The Panther Pathway enrichment analysis retrieved five significant pathways: blood coagulation, integrin signaling, Alzheimer disease-presenilin, angiogenesis and gonadotropin-releasing hormone receptor pathways (Table 3).

**Table 3:** Panther enriched pathways of the up-regulated differentially expressed genes (DEG) in the aggressive Lidia breed

Pathway	DEG	Fold Enrichment	P-value	FDR
Blood coagulation	10	4.32	2.51E-04	8.33E-03
Integrin signaling pathway	29	3.85	5.47E-09	4.54E-07
Alzheimer disease-presenilin pathway	18	3.67	7.66E-06	4.24E-04
Angiogenesis	21	3.15	1.18E-05	4.92E-04
Gonadotropin-realizing hormone receptor pathway	23	2.26	7.08E-04	1.96E-02

Within the down-regulated DEGs in the aggressive cohort, the GO biological processes included 260 genes as uniquely mapped IDs implicated in 243 processes (FDR ≤ 0.05), the highest significant values being dendritic cell cytokine production, trans-synaptic signaling by endocannabinoid, trans-synaptic signaling by lipid, negative regulation of renin secretion into blood stream and melanocyte adhesion, all with 84.4 fold enrichment and two genes associated with each process (Supplementary Table 5). The Panther enrichment pathway analysis retrieved two significant down-regulated pathways in the aggressive Lidia breed, both involved in two different types of Heterotrimeric G-protein signaling (Table 4).

**Table 4:** Panther Enriched pathways of the down-regulated DEG in the aggressive Lidia breed

Pathway	DEG	Fold Enrichment	P-value	FDR
Heterotrimeric G-protein signaling pathway-Gq alpha and Go alpha mediated pathway	8	5.32	1.96E-04	1.63E-02
Heterotrimeric G-protein signaling pathway-Gi alpha and Gs alpha mediated pathway	9	4.34	3.33E-04	1.84E-02

### Signaling networks and upstream regulators enrichment analysis

We used the IPA software to identify pathways to which the top DEGs (≥1 WR values) in common with the CSCA belong, as well as to explore the prediction of signaling networks connecting the DEGs.

Significant results are summarized in Supplementary Table 6. The most relevant results were obtained under the *physiological system development and function* and the *disease and disorders* categories. Within these categories, the top of the list gathered terms related with *Nervous system development and function* (highest *p*-value range of 4.10E-08 and 6 DEGs), and *Neurological disease* (highest p-value range of 6.33E-06 and 5 DEGs), and Psychological disorders (highest p-value range of 6.33E-06 and 3 DEGs) in their respective categories*.*

**Fig. 2:**
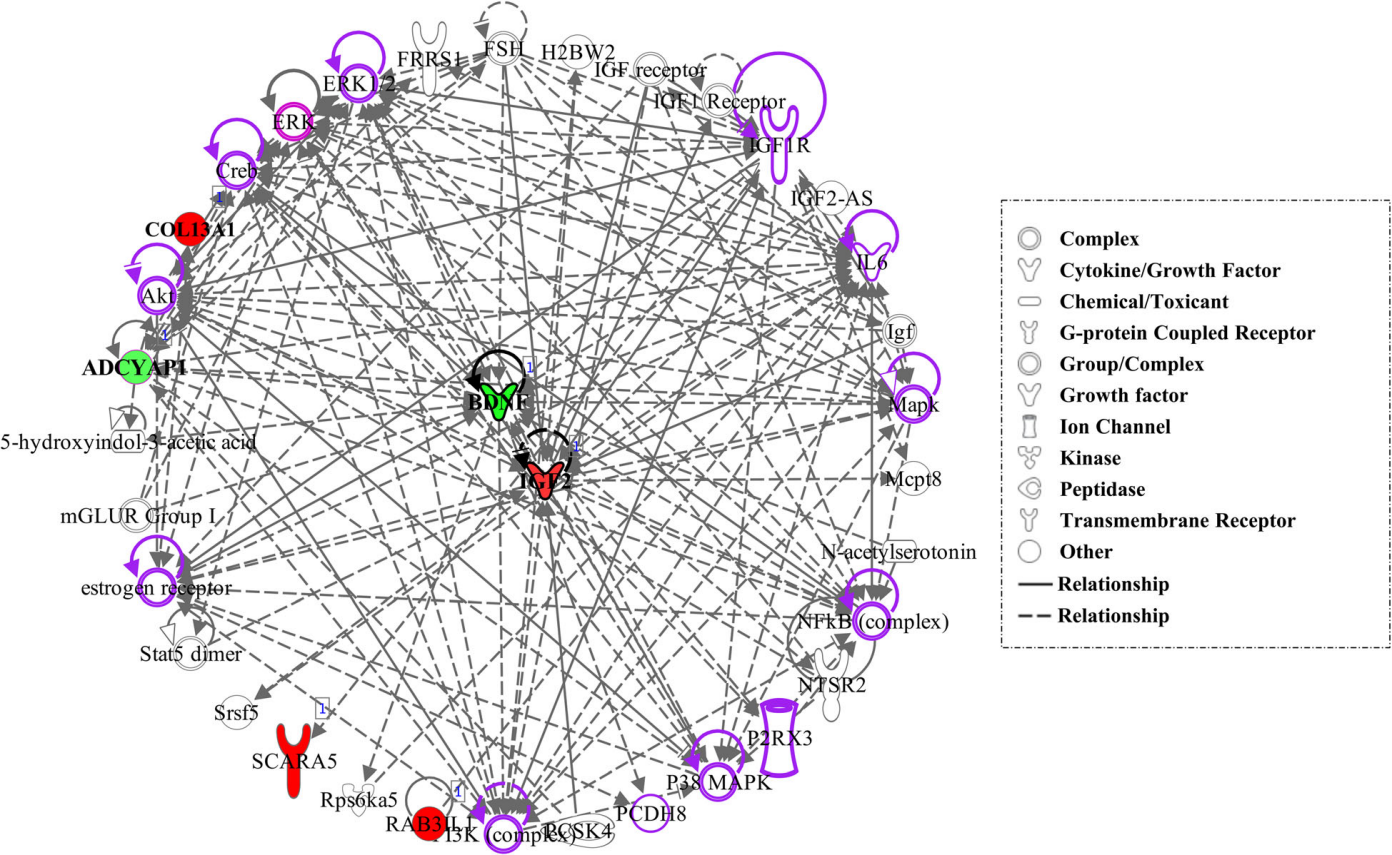
Top-scoring regulatory network identified with the IPA software, highlighting behavior related functions. Up and down-regulated differentially expressed genes (DEG) in the aggressive Lidia breed are displayed with red (up-regulated) and green (down-regulated) nodes, respectively. Genes are represented as nodes, and the molecular relationships between nodes are represented either as straight lines for direct interactions, or dotted lines for indirect interactions. The molecules highlighted in purple are those associated with the behavioral features detailed in Table 5

The top-scoring regulatory network predicted that 6 DEG; four up (*IGF2*, *COL13A1*, *RAB3IL1* and *SCARA5*) and two down-regulated (*ADCYAP1* and *BDNF*) in the aggressive cohort display interaction with 35 molecules. Two of those 6 DEGs, the up-regulated IGF2 and the down-regulated BDNF interact with most of the network’s molecules (Fig. 2). Furthermore, the functional network analyses predicted that 16 of these molecules are associated with behavioral function, among them *aggressive behavior* (*p*-value 2.99E-05) (Table 5).

**Table 5:** Regulatory network molecules involved in the category of *Diseases and Functions* associated with behavioral features

Diseases and Functions	Molecules	P-value	Genes
Emotional behavior	8	1.00 E-05	IGFR1, NFkB, estrogen receptor, Akt, *ADCYAP2, IL6*
Aggressive behavior	4	2.99E-05	*ADCYAP1, IL6, BDNF*, estrogen receptor
Hyperactive behavior	3	2.42E-05	*BDNF, ADCYAP1, IL6*
Depression-related behavior	2	2.71E-04	*BDNF, IL6*
Face Washing Behavior	1	5.29E-04	*ADCYAP1*

**Fig. 3:**
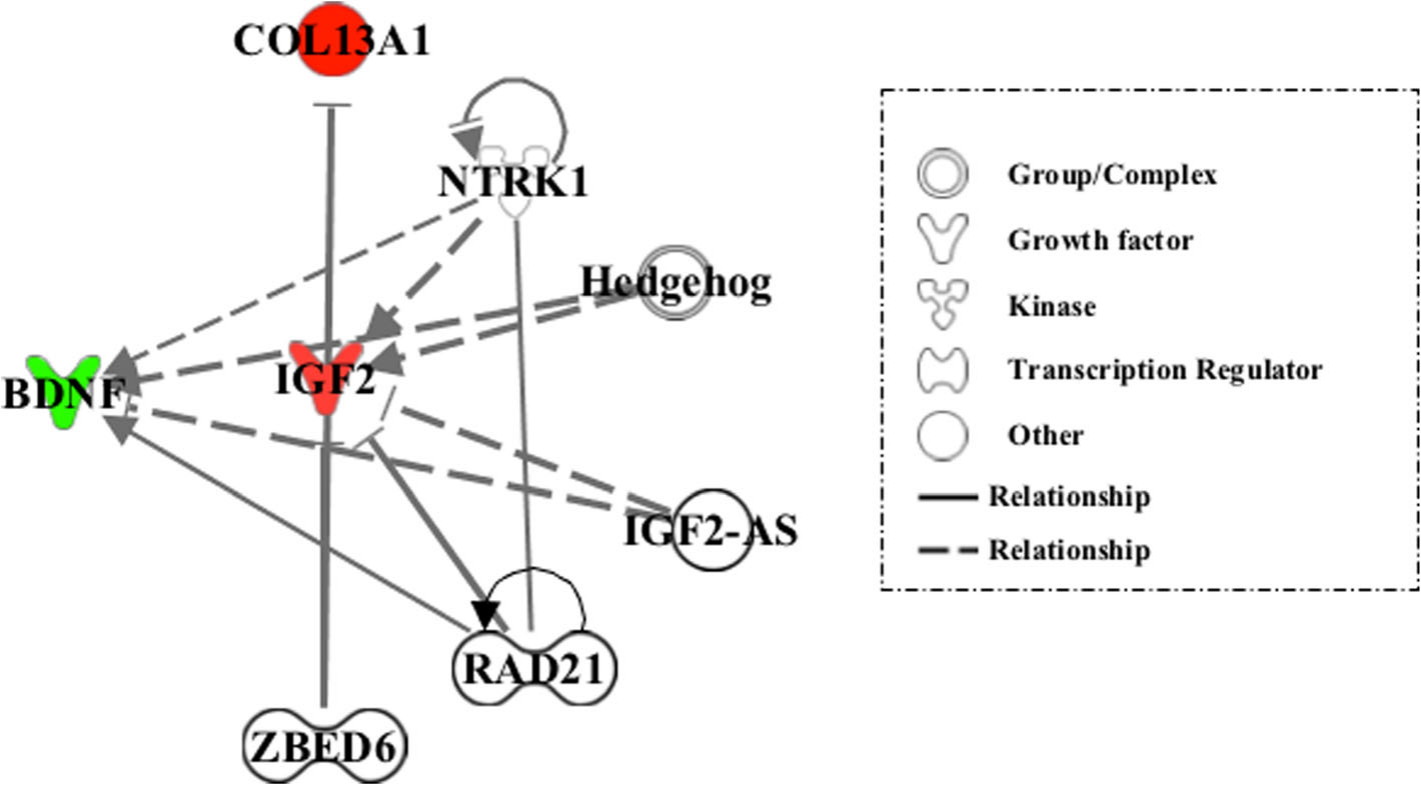
Major upstream regulators of the network of differentially expressed genes (DEG) in the aggressive Lidia group. There are five up-stream regulators predicted to be activated (underlined in black color). In red (up-regulated) and green (down-regulated) we can see the genes with expression changes in response to the activation of the upstream regulators. The shapes of the nodes represent the functional class of each gene or gene product, as defined in the legend. The straight and dashed lines represent direct and indirect interactions

Finally, the upstream analysis tool of the IPA package was used to identify the potential upstream regulators that may explain the differential patterns of expression between the up and down regulated DEGs in common with the CSCA in the aggressive cohort. By doing so, five main upstream regulators were identified: Insulin-Like Growth factor 2- Antisense RNA (IGF2-AS; p-value 2.53E-07), Neurotrophic Receptor Tyrosine Kinase 1 (*NTRK1; P*-value 2.32E-05), Zinc finger BED-Type Containing 6 (*ZBDE6;* p-value 4.71E-05), RAD21 Cohesin complex component (*RAD21;* p-value 5.58E-05), and Hedgehog (Hh; p-value 1.03E-04) (Fig. 3). All these genes, RNAs and proteins appear to be involved in a heterogeneous array of biological functions related to behavior development and cell-to-cell signaling interactions.

### Statistical analysis of aggression-associated differentially expressed genes (DEG)

In order to test whether the 50 DEGs with WR values of 1 or above identified in common with the CSCA represent a statistically significant association with aggressive behavior, we calculated the cumulative hypergeometric probability of this overlap occurring. Following removal of genes with no known orthologues in cattle from the list of aggression-associated genes, 1701 genes remained. Of these, 654 had a weighted ranking of 1 or above. Among the 1196 Lidia DEGs, 1157 had known one-to-one orthologues with humans, of which 50 were matches among the 654 genes with WR ≥ 1.

Given the estimated 22,000 genes in the bovine genome [44], the probability of there being 50 or more DEGs among the 654 aggression-associated genes was significantly above chance (*p* = 0.005). When restricting our analysis only to genes likely to be expressed in the cortex based on findings in other mammals—estimated at 85% of protein-coding genes in the genome [45] (18,700 genes in the case of cattle)—the probability of having 50 genes in our intersection was more likely to have occurred by chance (*p*-value = 0.07).

It could be considered that brain-expression studies of aggression in model animals (e.g. mouse, rat, and fox) are most similar in kind to our study. When we took only genes weight-ranked 1 or above that had been identified in previous expression studies (i.e. identified in at least two expression studies, or in one such study, as well as at least one GWAS, selective sweep, knock-out study, OMIM, or OMIA) 96 genes remained from our CSCA. Of these 13 were also present among the Lidia DEGs, a number significantly unlikely to have occurred by chance even under the restrictive analysis limiting our total genome population to the estimated 18,700 brain-expressed genes (*p*-value = 0.006). It should be noted that under more permissive analyses, where weighted ranking was not taken into account, all intersections between cattle DEGs and aggression-associated genes were significant, whether considering a genome population size of 22,000 or 18,700 genes, and whether considering all or only brain expression studies. These results confirm Lidia cattle as a valid model for the study of reactive aggression.

In the comparison with the high-frequency mutations and selective sweep studies in archaic humans, modern humans, and domesticated species (thirteen gene sets in total, compiled in [42], the only significant intersection was between the Lidia DEGs and genes with high frequency regulatory mutations in archaic compared to modern humans. 88 of the 1157 DEGs with known human orthologues were found among the 1003 genes with archaic high-frequency regulatory mutations (*p*-value = 0.0005, considering 18,700 as the total gene population size). This remained significant following Bonferroni correction for multiple comparisons (p-value = 0.007).

## Discussion

Understanding the complexity of the mechanisms behind the development of aggressive behaviors in humans and animals is still a challenge, although several molecular studies using different animal models have addressed this goal in recent years [16, 18, 24, 36–40, 46]. The present study represents the first description of transcriptional mechanisms affecting aggressive behavior in cattle.

The number of DEG identified in the bovine PFC (16,384) is similar to that identified in mice (15,423) [47] and silver foxes (14,000) [40], the latter also in the PFC. After correcting for Log_2_ Fold Change (FC), 918 up and 278 down-regulated genes displayed a wide array of functional pathways. Within the up-regulated enriched pathways in the aggressive cohort, we found biological functions related with processes such as cellular, muscular and SNC development and function, heart formation and development, and immune responses (Supplementary Table 5). Similar results were obtained by Kukekova et al. [24]; they compared the PFC expression between aggressive and docile strains of silver fox and also observed an enrichment of pathways related to cellular movement, growth and proliferation, hematological system development and antigen presentation.

Among the top enriched up-regulated pathways in the aggressive Lidia group, the integrin and Alzheimer disease-presenilin signaling pathways are well-known gene routes in the development of abnormal aggressive behaviors [48] (Table 3). In the nervous system, integrins are essential molecules for neuroplasticity, i.e. the ability to adapt to internal and external stimuli by reorganizing its structure, function and connections [49]. Increased expression of integrins contributes to imbalanced synaptic function in specific pathological conditions, such as Alzheimer disease and schizophrenia, both often accompanied by episodic aggression and violence [48]. It has been shown that aberrant presenilin expression also plays an important role in Alzheimer’s disease, with behaviors such as agitation and aggression frequently occurring in patients [22, 50].

Furthermore, the overexpression of genes belonging to the gonadotropin-realizing hormone (GnRH) receptor pathway may have a strong impact on the biological mechanisms leading to aggression. In boars, it has been observed that increased serum concentrations of GnRH result in higher levels of testosterone [51]. Testosterone is a sex hormone that has been implicated in the modulation of PFC activity; when increased, it may affect fear-processing circuitry, which has been associated with reactive and abnormal aggressive responses [52, 53].

Curiously, the up-regulated pathway showing the highest over-representation in the group of animals displaying agonistic behavior (4.32 fold enrichment), includes genes associated with blood coagulation. The links between the blood coagulation system and behavior are increasingly being recognized. For example, Yang et al. [54] observed a strong association of genes belonging to the blood coagulation pathway with human psychiatric disorders, such as major depression and suicidal behavior. The interrelation of hemostasis and angiogenesis, whereby the regulation of angiogenesis during vessel repair is mediated by proteins secreted by platelets [55], may explain the concomitant up-regulation of the angiogenesis pathway found here.

Regarding the down-regulated DEG detected in the group of aggressive animals, we found heterotrimeric G-protein pathways strongly suppressed (Table 2B). These routes are the main signaling pathways downstream of receptor activation and have been functionally associated with major depression and bipolar disorders [56]. The fighting reaction elicited in bulls in a *corrida* may temporarily antagonize the mechanisms implicated in low reactivity mental states, similar to those described in major depressive disorder [56].

To further disentangle the mechanisms activated by agonistic behaviors, we compared our dataset of DEG with those reported by Zhang-James et al. [34] in humans and mice, Kukekova et al. [24] in the silver fox, Eusebi et al. [16, 18] in cattle and Våge et al. [25] in dogs. As shown in Table 2, the level of concordance was low (50 genes in common were identified). Similarly, Zhang-James et al. [36] reported a modest gene overlap between different categories of genetic evidence (human GWAS, genes with known causal evidence and rodent transcriptome genes). According to these authors, the lack of overlap between studies suggests differences in the genetic etiology of aggression in different species and populations, and supports the complementarity of the gene sets detected. Nonetheless, the 50 genes we identified represented an above-chance intersection between Lidia DEG and the highest weighted aggression-associated genes in our CSCA.

All of the 24 up-regulated genes associated with aggressiveness and shared with previous studies, are essential for neurodevelopment. The highest weight-ranked gene was the Laminin Subunit Alpha 2 (*LAMA2)*, which encodes an extracellular matrix protein, and a mutation on which is associated with denervation atrophy of the muscle [57]. The D2 dopamine receptor (*DRD2*) was identified as one of the top ranked genes and has been widely studied in schizophrenia, for which SNPs located in the gene promoter affect its transcriptional activity [58]. Among the 26 down-regulated genes in common with the CSCA, a notable finding concerns the Glutamate Decarboxylase 2 (*GAD2*) one of our highly ranked genes which is considered a “top-down” modulator of aggressive acts, playing a pivotal role in the control systems deployed by the PFC to moderate agonistic reactions [59]. This gene, is a Gamma-aminobutyric acid (GABA)-synthesizing enzyme (converting glutamate to GABA), which has an inverse but linear relationship with measures of aggression: low levels of GABA in the anterior cingulate cortex are associated with high levels of aggression [59].

The downregulation of *GAD2* may contribute to a reversal of tameness or the maintenance/upregulation of wild-type aggression by targeting pathways typically implicated in the domestication process: Signals of selection across multiple domesticated species and in modern humans point to disproportionate targeting of metabotropic and kainite receptor genes that most often attenuate glutamatergic signaling. This has been proposed to alter the balance of glutamate-GABA interactions in stress-response circuits, including in prefrontal and limbic regions that regulate the hypothalamic-pituitary-adrenal (HPA) axis [9]. An attenuation of GABAergic signaling via downregulation of *GAD2* is likely to have the concomitant effect of altering inhibitory-excitatory balance in Lidia cattle. Further evidence for such alterations is suggested in the Panther GO enrichment analysis of downregulated DEG (Supplementary Table 5). The top enriched categories — regulation of amino acid import across plasma membrane (GO:0010958) and regulation of amino acid transmembrane transport (GO:1903789) — are each comprised of multiple genes that regulate the import of and uptake of glutamate (Supplementary Table 5). This is complemented by evidence of downregulated expression in Lidia cattle of the GABA-A receptor genes *GABRA3* and *GABRG2* as well *SLC17A7*, which encodes a vesicular glutamate transporter. Our observation that genes associated with archaic human regulatory changes show above-chance intersection with Lidia cattle DEGs mirrors findings that genetic changes in anatomically modern humans converge with those of domesticated species (including cattle) [9, 10]. Given that the relevant archaic genomic regions are implicated in the regulation of gene expression, our findings open up the intriguing possibility that the Lidia share aspects of their neurotranscriptomic and behavioral profile with archaic humans, including elevated stress and aggressive reactivity.

The analysis of the data with the IPA upstream enrichment tool retrieved one regulatory network related to diverse functions such as behavior, cellular movements and embryonic development. In the network shown in Fig. 2, two Mitogen Activated Protein Kinases (*MAPK*) and two Extracellular Signal Regulated Kinases (*ERK*) (outlined in grey color) occupy a position within the network. Similar results were obtained by Zhang-James et al. [36], who also identified ERK/MAPK signaling as mechanisms underlying aggression. Malki et al. [36] performed a genome-wide transcriptome analysis of mouse models of aggression and also observed that the MAPK signaling pathway was differentially expressed between the aggressive and non-aggressive lines. The *MAPK/ERK* cascade is a key regulator of cell growth and proliferation, but most important, this signaling pathway activates the binding of different integrins at the cell surface to extracellular matrix proteins [60], linking its function with the up-regulated integrin pathway explained above. This pathway is also highlighted as having been altered in multiple domestication events (including that of cattle), as well as in modern-human evolution [10].

Finally, five upstream regulators were predicted to be major transcriptional regulators of a set of three DEGs; two up-regulated (*COL13A1*and *IGF2*) and the down-regulated *BDNF* gene (Fig. 3). The modulator effect of these molecules appears to increase the up-regulation of biological processes such as hyperactive behavior and anxiety, which are often associated with aggressiveness, as well Alzheimer disease, in concordance with the above findings. We also found that the upstream regulators promote an increase in nociception. Although distinct from aggressive reactivity, an enhancement in the capacity of Lidia cattle to respond to potentially damaging stimuli may promote the display of aggressive behaviors.

## Conclusions

This the first time that a comparison of the differences in genomic expression between aggressive and non-aggressive selected cattle breeds has been performed, identifying 918 up and 278 down-regulated genes in the PFC. We have also undertaken a cross-species comparison analysis to identify genes in common implicated in aggressiveness and to investigate their regulatory networks. Our results include up-regulation in the aggressive cohort of pathways such as the Alzheimer disease-presenilin, integrins and the ERK/MAPK signaling cascade, all routes implicated in the development of abnormal aggressive behaviors and neurophysiological disorders. We also identified normal mechanisms enhancing aggression across species such us the up-regulation of gonadotropins and, hence, testosterone, whose levels have been widely linked with agonistic reactions. In contrast, heterotrimeric G-protein pathways, previously associated with low reactivity mental states like those involved in major depression, or the *GAD2* gene, which plays a pivotal role in the control systems deployed by the PFC to repress agonistic reactions, are both down-regulated, guaranteeing the development of the adequate combative responses needed during a “*corrida*” event. Nonetheless, despite the PFC being a key region for the modulation of aggressive behavior, it may not be representative of other brain regions also reported to play important roles in aggression, such as the amygdala, hippocampus or hypothalamus. Furthermore, although our findings are consistent with gene expression studies of aggressive behaviors in other species, one limitation of the current study is that we still need to consider some inherent biasing factors such as those associated to breed differences and external factors such as the conditions of the animal’s slaughter.

Nevertheless, this study constitutes an important first step towards the identification of genes that promote aggression in cattle, and provides a novel species as model organism for disentangling the variable mechanisms underlying aggressive behaviors in our own species.

## Supplementary Information

**Additional file 1: Table S1.** Gene-list of the different studies containing the total number of genes associated with aggressive behavior across gene sets. **Table S2.**:= Lists of Significant genes in human evolution (from Zanella et al. 2019)**. Table S3.** Summary statistics per sample of the RNA-seq analysis of aggressive (Lidia breed) and non-aggressive (Wagyu breed) groups. **Table S4.** Gene list report on the 918 up-regulated and 278 down-regulated (0.1 ≤ Log2FC ≤ 0.1) sequences in the aggressive Lidia breed. **Table S5.** List of the significantly overrepresented Ingenuity Pathway Analysis (IPA) gene ontology terms for the three main family categories in the up and down-regulated genes in the aggressive group. **Table S6.** Regulatory network of genes differentially expressed (*P*-value ≤ 0.01 ) in up and down regulated differentially expressed genes (DEG) in the aggressive Lidia breed.

**Additional file 2: Figure S1.** Anatomical location of the medial prefrontal cortex (PFC) dissection samples.

## Data Availability

Illumina reads generated from all samples have been deposited in the NCBI GEO / bioproject browser database (Accession Number: GSE148938).

## Abbreviations

PFC: Prefrontal cortex
DEG: Differentiated expressed genes
CSCA: Cross-species comparative analysis
GWAS: Genome-wide association studies
OMIM: Online Mendelian Inheritance in Man database
KO: Knockout mice report
OMIA: Online Mendelian Inheritance in Animals
WR: Weighted ranking
FC: Fold Change

## References

[1] Tremblay RE, Nagin D, Tremblay RE, Hartup WW, Archer J (2008). The developmental origins of physical aggression: developmental origins of aggression.

[2] Haller J, Tóth M, Halasz J, De Boer SF (2006). Patterns of violent aggression-induced brain c-fos expression in male mice selected for aggressiveness. Physiol Behav.

[3] Manchia M, Fanos V (2017). Targeting aggression in severe mental illness: the predictive role of genetic, epigenetic, and metabolomics markers. Prog Neuro-Psychopharmacol Biol Psychiatry.

[4] Tuvblad C, Baker LA (2011). Human aggression across the lifespan: genetic propensities and environmental moderators. Adv Genet.

[5] Blanchard DC, Blanchard RJ (2003). What can animal aggression research tell us about human aggression?. Horm Behav.

[6] de Boer SF, Van der Vegt BJ, Koolhaas JM (2003). Individual variation in aggression of feral rodent strains: a standard for the genetics of aggression and violence?. Behav Genet.

[7] McGuire J (2008). A review of effective interventions for reducing aggression and violence. Philos Trans R Soc Lond Ser B Biol Sci.

[8] Hare B, Wobber V, Wrangham R (2012). The self-domestication hypothesis: evolution of bonobo psychology is due to selection against aggression. Anim Behav.

[9] O’Rourke T, Boeckx C (2020). Glutamate receptors in domestication and modern human evolution. Neurosci Biobehav Rev.

[10] Theofanopoulou C, Gastaldon S, O’Rourke T, Samuels BD, Messner A, Martins PT (2017). Self-domestication in *Homo sapiens*: Insights from comparative genomics. PLoS One.

[11] Sánchez-Villagra MR, van Schaik CP (2019). Evaluating the self-domestication hypothesis of human evolution. Evol Anthropol.

[12] Trut L, Oskina I, Kharlamova A (2009). Animal evolution during domestication: the domesticated fox as a model. Biol Essays.

[13] Wrangham RW (2019). Hypotheses for the evolution of reduced reactive aggression in the context of human self-domestication. Front Psychol.

[14] Silva B, Gonzalo A, Cañón J (2006). Genetic parameters of aggressiveness, ferocity and mobility in the fighting bull breed. Anim Res.

[15] Menéndez-Buxadera A, Cortés O, Cañon J (2017). Genetic (co) variance and plasticity of behavioural traits in Lidia bovine breed. Ital J Anim Sci.

[16] Eusebi PG, Cortés O, Dunner S, Cañón J (2018). Detection of selection signatures for agonistic behavior in cattle. J Anim Breed Genet.

[17] Craig IW, Halton KE (2009). Genetics of human aggressive behaviour. Hum Genet.

[18] Eusebi PG, Sevane N, Cortés O, Contreras E, Cañon J, Dunner S (2019). Aggressive behavior in cattle is associated with a polymorphism in the *MAOA* gene promoter. Anim Genet.

[19] Mariadassou M, Ramayo-Caldas Y, Charles M, Féménia M, Renand G, Rocha D (2020). Detection of selection signatures in Limousin cattle using whole-genome resequencing. Anim Genet.

[20] Qanbari S, Pausch H, Jansen S, Somel M, Strom TM, Fries, et al. Classic Selective Sweeps Revealed by Massive Sequencing in Cattle. PLoS Genet. 2014;10(2). 10.1371/journal.pgen.1004148 PMC3937232 24586189

[21] Miczek KA, de Almeida RM, Kravitz EA, Rissman EF, de Boer SF, Raine A (2007). Neurobiology of escalated aggression and violence. J Neurosci.

[22] Siever LJ (2008). Neurobiology of aggression and violence. Am J of Psych.

[23] Brower MC, Price BH (2001). Neuropsychiatry of frontal lobe dysfunction in violent and criminal behaviour: a critical review. J Neurol Neurosurg Psychiatry.

[24] Kukekova AV, Johnson JL, Teiling C, Li L, Oskina IN, Kharlamova AV, Gulevich RG, Padte R, Dubreuil MM, Vladimirova AV, Shepeleva DV, Shikhevich SG, Sun Q, Ponnala L, Temnykh SV, Trut LN, Acland GM (2011). Sequence comparison of prefrontal cortical brain transcriptome from a tame and an aggressive silver fox (Vulpes vulpes). BMC Genomics.

[25] Våge J, Bønsdorff TB, Arnet E, Tverdal A, Lingaas F (2010). Differential gene expression in brain tissues of aggressive and non-aggressive dogs. BMC Vet Res.

[26] Takanishi N, Oishi K, Kumagai H, Uemura M, Hirooka H (2015). Factors influencing the priority of access to food and their effects on the carcass traits for Japanese black (Wagyu) cattle. Animal..

[27] Boletin Oficial del Estado. 2001 Real Decreto 60/2001, de 26 de enero, sobre prototipo racial de la raza bovina de lidia. Boletin Oficial del Estado 38, 5255–5261.

[28] Domecq JP (2009). Del toreo a la bravura.

[29] Lomillos JM, Alonso ME (2019). Revisión de la alimentación de la raza de lidia y caracterización de las principales patologías asociadas al cebo del toro en la actualidad. ITEA Informacion Tecnica Economica Agraria.

[30] Gotoh T, Nishimura T, Kuchida K, Mannen H (2018). The Japanese Wagyu beef industry: current situation and future prospects—a review. Asian Australas J Anim Sci.

[31] Schmieder R, Edwards R (2011). Quality control and preprocessing of metagenomic datasets. Curr Bioinforma.

[32] Dobin A, Davis CA, Schlesinger F, Drenkow J, Zaleski C, Jha S, Batut P, Chaisson M, Gingeras TR (2013). STAR: ultrafast universal RNA-seq aligner. Curr Bioinforma.

[33] Li H, Handsaker B, Wysoker A, Fennell T, Ruan J, Homer N (2010). 1000 genome project data process-ing subgroup. The sequence alignment/map format and SAMtools. Curr Bioinforma.

[34] Trapnell C, Williams BA, Pertea G, Mortazavi A, Kwan G, Van Baren MJ (2010). Transcript assembly andquantification by RNA-Seq reveals unannotated transcripts and isoform switching during cell differentiation. Nat Biotechnol.

[35] Goff L, Trapnell C, Kelley D (2012). cummeRbund: Analysis, exploration, manipulation, and visualization of Cufflinks high-throughput sequencing data. R package version 2.6.1.

[36] Zhang-James Y, Fernàndez-Castillo N, Hess JL, Malki K, Glatt SJ, Cormand B, Faraone SV (2019). An integrated analysis of genes and functional pathways for aggression in human and rodent models. Mol Psych.

[37] Fernández del Castillo N, Cormand B (2016). Aggressive behavior in humans: genes and pathways identified through association studies. Am J Med Genet.

[38] Malki K, Pain O, Du Rietz E, Tosto MG, Paya-Cano J, Sandnabba KN (2014). Genes and gene networks implicated in aggression related behaviour. Neurogenetics..

[39] Clinton SM, Stead JD, Miller S, Watson SJ, Akil H (2011). Developmental underpinnings of differences in rodent novelty-seeking and emotional reactivity. Eur J Neurosci.

[40] Kukekova AV, Johnson JL, Xiang X, Feng S, Liu S, Rando HM, Kharlamova AV, Herbeck Y, Serdyukova NA, Xiong Z, Beklemischeva V, Koepfli KP, Gulevich RG, Vladimirova AV, Hekman JP, Perelman PL, Graphodatsky AS, O’Brien SJ, Wang X, Clark AG, Acland GM, Trut LN, Zhang G (2018). Red fox genome assembly identifies genomic regions associated with tame and aggressive behaviours. Nat Ecol Evol.

[41] Durinck S, Moreau Y, Kasprzyk A, Davis S, De Moor B, Brazma A (2005). BioMart and bioconductor: a powerful link between biological databases and microarray data analysis. Curr Bioinforma.

[42] Zanella M, Vitriolo A, Andirko A, Martins PT, Sturm S, O’Rourke T, Laugsch M (2019). Dosage analysis of the 7q11. 23 Williams region identifies BAZ1B as a major human gene patterning the modern human face and underlying self-domestication. Sci Adv.

[43] Krämer A, Green J, Pollard J, Tugendreich S (2014). Causal analysis approaches in ingenuity pathway analysis. Curr Bioinforma.

[44] Elsik CG, Tellam RL, Worley KC (2009). The genome sequence of taurine cattle: a window to ruminant biology and evolution. Science..

[45] Sjöstedt E, Zhong W, Fagerberg L, Karlsson M, Mitsios N, Adori C, Oksvold P, Edfors F, Limiszewska A, Hikmet F, Huang J, Du Y, Lin L, Dong Z, Yang L, Liu X, Jiang H, Xu X, Wang J, Yang H, Bolund L, Mardinoglu A, Zhang C, von Feilitzen K, Lindskog C, Pontén F, Luo Y, Hökfelt T, Uhlén M, Mulder J (2020). An atlas of the protein-coding genes in the human, pig, and mouse brain. Science..

[46] Veroude K, Zhang-James Y, Fernandez-Castillo N, Bakker MJ, Cormand B, Faraone SV (2016). Genetics of aggressive behavior: an overview. Am J Med Genet B.

[47] Han X, Wu X, Chung WY, Li T, Nekrutenko A, Altman NS, Chen G, Ma H (2009). Transcriptome of embryonic and neonatal mouse cortex by high-throughput RNA sequencing. Proc Natl Acad Sci.

[48] Wu X, Reddy DS (2012). Integrins as receptor targets for neurological disorders. Pharmacol Ther.

[49] Cramer SC, Sur M, Dobkin BH, O'brien C, Sanger TD, Trojanowski JQ (2011). Harnessing neuroplasticity for clinical applications. Brain..

[50] Ballard CG, Gauthier S, Cummings JL, Brodaty H, Grossberg GT, Robert P, Lyketsos CG (2009). Management of agitation and aggression associated with Alzheimer disease. Nat Rev Neurol.

[51] Wise T, Zanella EL, Lunstra DD, Ford JJ (2010). Relationships of gonadotropins, testosterone, and cortisol in response to GnRH and GnRH antagonist in boars selected for high and low follicle-stimulating hormone levels. J Anim Sci.

[52] Radke S, Volman I, Mehta P, van Son V, Enter D, Sanfey A, Toni I (2015). Testosterone biases the amygdala toward social threat approach. Sci Adv.

[53] Bakker-Huvenaars MJ, Greven CU, Herpers P, Wiegers E, Jansen A, Van Der Steen R (2018). Saliva oxytocin, cortisol, and testosterone levels in adolescent boys with autism spectrum disorder, oppositional defiant disorder/conduct disorder and typically developing individuals. Eur Neuropsychopharmacol.

[54] Yang Y, Chen J, Liu C, Fang L, Liu Z, Guo J, Cheng K, Zhou C, Zhan Y, Melgiri ND, Zhang L, Zhong J, Chen J, Rao C, Xie P (2016). The extrinsic coagulation pathway: a biomarker for suicidal behavior in major depressive disorder. Sci Rep.

[55] Browder T, Folkman J, Pirie-Shepherd S (2000). The hemostatic system as a regulator of angiogenesis. J Biol Chem.

[56] González-Maeso J, Meana JJ (2006). Heterotrimeric g proteins: insights into the neurobiology of mood disorders. Curr Neuropharmacol.

[57] Hall TE, Bryson-Richardson RJ, Berger S, Jacoby AS, Cole NJ, Hollway G (2007). The zebrafish candyfloss mutant implicates extracellular matrix adhesion failure in laminin alpha2-deficient congenital muscular dystrophy. Proc Natl Acad Sci.

[58] d'Souza UM, Kel A, Sluyter F (2003). From transcriptional regulation to aggressive behavior. Behav Genet.

[59] Jager A, Amiri H, Bielczyk N, van Heukelum S, Heerschap A, Aschrafi A (2017). Poelmans G, et al cortical control of aggression: GABA signalling in the anterior cingulate cortex. Eur Neuropsychopharmacol.

[60] Yee KL, Weaver VM, Hammer DA (2008). Integrin-mediated signalling through the MAP-kinase pathway. IET Syst Biol.

