# Supplementary material for: Gene expression profiles underlying aggressive behavior in the prefrontal cortex of cattle
Source: BMC Genomics. 2021 Apr 7;22:245. doi: 10.1186/s12864-021-07505-5 (PMC8028707; doi:10.1186/s12864-021-07505-5)
Supplement: Supplementary file 2 — Additional file 2: Figure S1. Anatomical location of the medial prefrontal cortex (PFC) dissection samples. [file 12864_2021_7505_MOESM2_ESM.pdf]

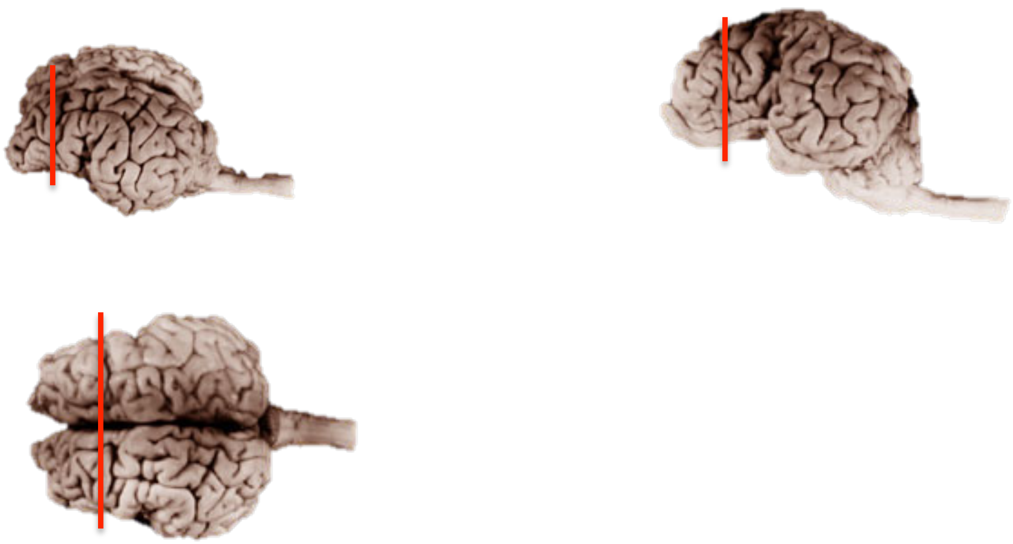

Figure S1 Anatomical location of the medial prefrontal cortex (PFC) dissection samples.

Copyright images from http://neurosciencelibrary.org/
